# Limited evidence to recommend lactate kinetics-guided therapy

**DOI:** 10.1186/s13054-017-1752-8

**Published:** 2017-07-03

**Authors:** Daniel O. Thomas-Rüddel, Michael Bauer

**Affiliations:** Department of Anesthesiology and Intensive Care Therapy and Center for Sepsis Control and Care, Jena University Hospital, Am Klinikum 1, 07747 Jena, Germany

We congratulate Zhou and colleagues on their randomized trial comparing central venous oxygen saturation (ScvO2)- and lactate-driven resuscitation in septic shock [1] and would like to offer the following comments.

Only the use of inotropes and blood transfusions were triggered by lactate kinetics or ScvO2 according to the reported treatment algorithms. There was no difference in those treatments; therefore, it does not seem plausible to attribute the observed huge differences in mortality to the different treatment algorithms. A fragility index [2] of 1 indicates a considerable risk that the observed difference was a chance finding, not surprising as the trial was powered for non-inferiority and therefore had a quite small sample size. The findings should also be interpreted in the light of three recent trials that could show no survival benefit for ScvO2-driven early goal-directed therapy [3].

Fluid therapy was guided by the same central venous pressure (CVP) target (8–12 mmHg) as the first step of the algorithm in both groups but significantly higher volumes were infused in the lactate-guided group. This was associated with slightly higher CVP values during the first 12 h of resuscitation. Looking at the reported median CVP values one recognizes that only approximately 50% of all patients reached the targeted CVP levels at any given time point. This is much lower than the reported algorithm compliance of 90%. One might hypothesize that the elevated lactate encouraged fluid therapy and possibly other unmeasured diagnostic and therapeutic steps, while in the context of mostly normal ScvO2 levels even the first step of the algorithm was less strictly followed, resulting in performance bias.

In the trial by Jansen et al. [4] a higher use of fluids, blood products, inotropes, and vasodilators was observed in the lactate-guided group during the early phase of resuscitation. This was associated with a lower mortality in adjusted analysis but not with any differences in lactate kinetics between groups. It would be of high interest if Zhou and colleagues could provide lactate kinetics over time for both groups. Furthermore, elevated lactate levels in sepsis have multiple causes apart from poor tissue perfusion [5].

In our opinion an initial elevation or no reduction of lactate levels in sepsis indicates patients at high risk for negative outcomes. It should trigger immediate attention, diagnostic workup, and frequent reevaluation of therapies, but no specific algorithm can be recommended based on the available evidence.

## Abbreviations

CVP: Central venous pressure
ScvO2: Central venous oxygen saturation
